# Aquatic methane dynamics in a human‐impacted river‐floodplain of the Danube

**DOI:** 10.1002/lno.10346

**Published:** 2016-06-20

**Authors:** Anna Katarzyna Sieczko, Katalin Demeter, Gabriel Andreas Singer, Michael Tritthart, Stefan Preiner, Magdalena Mayr, Karin Meisterl, Peter Peduzzi

**Affiliations:** ^1^ Department of Limnology and Bio‐OceanographyUniversity of ViennaViennaAustria; ^2^ Department of EcohydrologyLeibniz‐Institute of Freshwater Ecology and Inland FisheriesBerlinGermany; ^3^ Department of Water, Atmosphere and EnvironmentUniversity of Natural Resources and Life SciencesViennaAustria; ^4^ WasserCluster Lunz GmbHDr. Carl Kupelwieser Promenade 5Lunz am SeeAustria; ^5^ University of Natural Resources and Life Sciences, Institute of Hydrobiology and Aquatic Ecosystem ManagementViennaAustria

## Abstract

River‐floodplain systems are characterized by changing hydrological connectivity and variability of resources delivered to floodplain water bodies. Although the importance of hydrological events has been recognized, the effect of flooding on CH_4_ concentrations and emissions from European, human‐impacted river‐floodplains is largely unknown. This study evaluates aquatic concentrations and emissions of CH_4_ from a highly modified, yet partly restored river‐floodplain system of the Danube near Vienna (Austria). We covered a broad range of hydrological conditions, including a 1‐yr flood event in 2012 and a 100‐yr flood in 2013. Our findings demonstrate that river‐floodplain waters were supersaturated with CH_4_, hence always serving as a source of CH_4_ to the atmosphere. Hydrologically isolated habitats in general have higher concentrations and produce higher fluxes despite lower physically defined velocities. During surface connection, however, CH_4_ is exported from the floodplain to the river, suggesting that the main channel serves as an “exhaust pipe” for the floodplain. This mechanism was especially important during the 100‐yr flood, when a clear pulse of CH_4_ was flushed from the floodplain with surface floodwaters. Our results emphasize the importance of floods differing in magnitude for methane evasion from river‐floodplains; 34% more CH_4_ was emitted from the entire system during the year with the 100‐yr flood compared to a hydrologically “normal” year. Compared to the main river channel, semi‐isolated floodplain waters were particularly strong sources of CH_4._ Our findings also imply that the predicted increased frequency of extreme flooding events will have significant consequences for methane emission from river‐floodplain systems.

Recent global estimates on the areal extent of inland waters are greater than previous appraisals (Downing et al. 2012; Verpoorter et al. 2014). This was also linked to higher global estimates of CO_2_ evasion (Battin et al. 2008; Raymond et al. 2013). Methane (CH_4_) is recognized as the second most important anthropogenic greenhouse gas (GHG), with a global warming potential 25 times that of CO_2_, yet much less is known about the emissions of this trace gas from inland waters (Bastviken et al. 2011). Regarding flowing waters, existing research has focused mostly on the emissions from large rivers and is associated with high uncertainty. Downing et al. (2012) emphasized that the contribution of rivers and streams may be +20 to +200% greater than previous estimates. The role of fringing floodplains and wetlands and their contribution to global methane emissions have received some attention (Aselmann and Crutzen 1989; Sha et al. 2011). Shallow lakes and wetlands are important for the Earth's carbon balance, but the majority of existing research covers emissions from large river‐floodplain systems such as the Amazon (Melack et al. 2004; Ringeval et al. 2014) or the Pantanal (Marani and Alvala 2007; Bastviken et al. 2010). More recently, some attention has been given to small wetlands because they possibly contribute more to global methane emissions than previously considered (Yavitt 2010).

Due to variable hydrology and long‐term retention between floods, river‐floodplain systems possess ample opportunities to store and process carbon (Battin et al. 2008). Although some studies consider river‐floodplains to be carbon neutral (Batson et al. 2015), most identify them as a source of CH_4_ to the atmosphere (Denman et al. 2007; Sawakuchi et al. 2014). Saarnio et al. (2009), for example, evaluated total CH_4_ emissions from European floodplains and wetlands in a meta‐analysis, yet their estimates bear considerable uncertainties due to the small number of studies and high variations in CH_4_ emission. One factor that potentially greatly affects CH_4_ emissions is hydrological disturbance (Altor and Mitsch 2008; Gatland et al. 2014). Changing hydrology, including seasonal flooding events, results in large temporal changes in methane evasions from floodplains (Otter and Scholes 2000). Nonetheless, the importance of hydrology, especially the occurrence of extreme hydrological events, for CH_4_ evasion from European river‐floodplains has not received significant attention. An often overlooked, key aspect is the changing area of the inundated floodplain: it must be taken into account when estimating total evasion from such systems (Smith et al. 2000).

Due to river regulation, damming, construction of hydroelectric power stations, agriculture and other anthropogenic activities, the areas of many European floodplains have been reduced significantly (Klimo and Hager 2001), with substantial implications for floodplain functions. There is evidence that human impacts are responsible for increased methane fluxes from wetlands (Petrescu et al. 2015), but the study of Frolking et al. (2011) implies that anthropogenic disturbance may also result in reduced CH_4_ emissions. This suggests that anthropogenic degradation of floodplain systems can influence their overall carbon flux. In this light, a potential human impact on CH_4_ emissions from modified river‐floodplains, including restored sections, remains unclear. Considering that much of Europe's floodplains have been degraded, the significance for CH_4_ emissions must be considered.

In this study, we assess CH_4_ emissions from a human‐modified and from a partly restored river‐floodplain section of the Danube near Vienna (Austria). We investigated whether hydrological events differing in magnitude affect the concentration and the evasion of CH_4_ and hypothesized that floodplain waters are important sources of CH_4_ in comparison to the main river channel. Finally, we discuss possible consequences of variously intense anthropogenic alterations of river‐floodplain systems on CH_4_ emissions.

## Materials and methods

### Study area and sampling design

**Figure 1:**
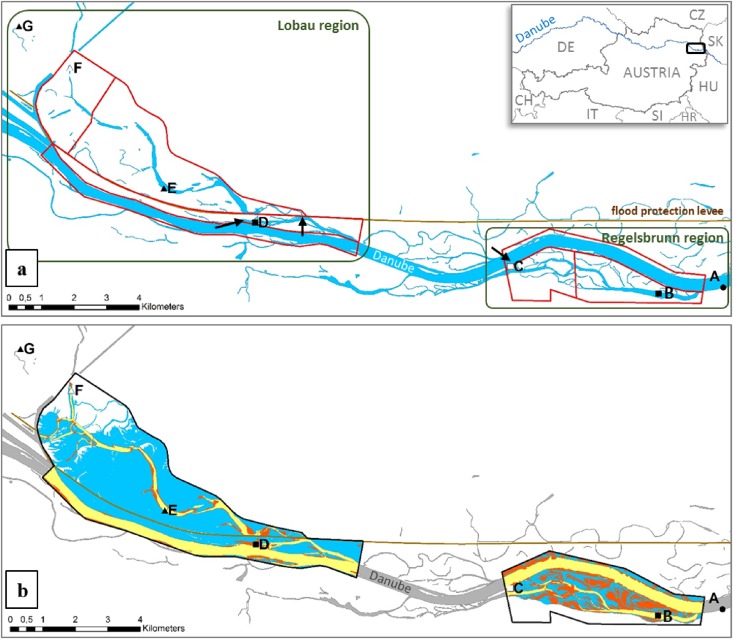
The Danube River and its floodplains downstream of Vienna. The small map shows Austria. (**a**) The letters indicate the sampling stations. Regelsbrunn and Lobau regions indicate different subsystems of the river‐floodplain and the arrows point at the inlet where the flooding water enters these floodplain areas. Floodplain sections used for upscaling are marked with the red line. (**b**) The aerial extent of mean water (yellow), a 1‐yr flood (red) and a 100‐hundred‐yr flood (blue) in the two studied regions.

Our study was conducted in a river‐floodplain system located in the Danube Floodplain National Park downstream of Vienna (Austria, river km 1895–1918). Throughout the centuries, this area has been severely impacted by human alterations, considerably decreasing the active flood plain area (Hein et al. 2016). Notably, levees built during the19th century transformed lotic floodplain water bodies into lentic side‐arms (Hohensinner et al. 2013). In recent decades, however, some floodplain sections have been successfully restored with the aim to re‐establish conditions typical for “active,” natural floodplain systems. Thereby, near‐natural‐river‐floodplain sections were recreated close to the town Regelsbrunn and in parts close to the area Lobau (Fig. 1) (Schiemer et al. 1999; Schiemer et al. 2006), where frequent surface connection with the main channel (∼200 d per year) now occurs. Most parts of Lobau, however, are still heavily protected from direct through‐flow (Fig. 1) because only a small downstream opening allows surface connection at higher water flow (Reckendorfer and Hein 2000). Lobau is thus characterized by dampened water level fluctuations (Schiemer et al. 1999) in a number of lentic backwaters, that are only occasionally connected by back‐flowing floods (Reckendorfer et al. 2013). There are also completely disconnected parts of Lobau that formerly belonged to the active floodplain but now never experience surface connection to the Danube. Groundwater connection with the Danube, however, does exist (Griebler and Mösslacher 2003). However for this complex processes no detailed data are available so far.

For this study, we selected sites that represent a gradient of connectivity to the main channel. They ranged from isolated (station G, connected 0 d yr^−1^), through semi‐isolated (station F and E, connected 0 −20 and 20–180 d yr^−1^, respectively) to dynamic (station B, C, D, connected > 180 d yr^−1^) (Fig. 1a). In dynamic water bodies (Fig. 1, stations B, C, D), lotic conditions can be frequently observed, whereas semi‐isolated E and F are open‐water backwaters mainly characterized by lentic conditions. The completely disconnected, isolated station G is comparably small, lake‐like, highly sheltered and shaded. It was once part of the former, active floodplain, but because of the river regulation in the 19th century it is now never connected to the Danube, even during extreme flooding events.

We performed two sampling campaigns, one in 2012 (Apr–Dec) and one in 2013 (Apr‐Aug), each covering a spring pre‐flood, early summer flood and a post‐flood hydrological phase. At the Austrian stretch the Danube receives large amounts of snowmelt and glacial runoff from its southern tributaries and its flow regime is therefore characterized by predictably high water levels in early summer (Heiler et al. 1995). Sampling frequency was adaptive, either bi‐weekly or monthly in the pre‐ and post‐flood phase, and increased to 2–3 times per week at the onset of a flood.

### Hydrology and aquatic surface area

The Danube discharge and water level data were provided by the Hydrological Office of Lower Austria. They were obtained from station A located downstream of the floodplain. Mean water, 1‐yr and 100‐yr flooding events are defined by discharges of 1930 m^3^ s^−1^, 5300 m^3^ s^−1^, and 10,500 m^3^ s^−1^, respectively (www.noel.gv.at). Flow velocity in the main channel was calculated from the discharge time series and cross section data (“Via Donau”‐company, unpublished data).

Stations B and C are located in a hydrologically different area than stations D through F. While the latter stations are subject to backwater flow only—as inflow and outflow take place through a single cross section—the first two are located in a river subsystem generally characterized by through‐flow, hence characterized by separate boundary conditions for inflow and outflow. Therefore, different model approaches were selected for these two regions. For stations B and C, flow velocities were obtained from the model by Reckendorfer and Steel (2004), and water levels were computed from gauge readings taken during each sampling campaign. For stations D, E and F, flow velocities were acquired from the hydrological model of Gabriel et al. (2015), and water levels were calculated for the discharges observed at sampling dates with the hydrodynamic model of Tritthart et al. (2011) developed for the Lobau area. The three referenced models are comparable in the quality of their output parameters because all of them were calibrated and validated successfully on measured hydrographs at several gauges located within the study site. As flow velocity follows directly from an unsteady continuity condition in the river subsystems investigated, it is not expected to be associated with any larger error than the water surface, independent of the model used.

For each sampled water body and sampling time point, the volume and surface area of the flooded region were calculated based on the water level and the digital elevation model (DEM) (Via Donau, unpublished data, resolution: 2.5 × 2.5 m), using the “3D Analyst Tools” in ArcMap 10.1 (ESRI). The average depth of the water body was obtained by dividing the volume by the surface area of the water body. These values were used to calculate CH_4_ flux.

### Sampling and gas chromatography

Temperature, oxygen saturation (%), pH, and conductivity were determined *in situ* (WTW Oxi 330, WTW pH 330 and WTW Cond 330, respectively). Water and air samples from all stations were collected in triplicate in 50 mL serum bottles to measure CH_4_ concentrations. Water samples were collected by filling bottles without headspace and closing them with crimp‐seal gas‐tight rubber stoppers under water. Bottles were prepared with a small amount of precipitated sodium azide (NaN_3_, final concentration 0.54 mol L^−1^) for preservation prior to sampling. Samples were stored at 4°C pending analysis by gas chromatography (Agilent GC 6890N equipped with a flame ionization detector and an automatic gas‐injection unit). The GC sampled a headspace that was created by replacing 15–20% of sample water with CH_4_‐free N_2_ and equilibrated with the water phase at a controlled temperature (30°C water bath placed on a rotary shaker); exact headspace volume was determined by mass difference. Equilibration pressure was recorded automatically by the gas‐injection unit (Joint Analytical Systems) sampling the headspace. One CH_4_‐standard (50 ppm) was analyzed prior to each batch of 15 samples. The CH_4_ concentration in the original water sample (CH_4_w [*μ*mol L^−1^]) was determined based on Henry's law using equilibration pressure and temperature, and water and headspace volume (Sander 2014). Field partial pressure of CH_4_ was then computed based on Henry's law from concentration and using field water temperature and atmospheric pressure sourced from close‐by meteorological stations (Austrian Central Institute for Meteorology and Geodynamics, ZAMG).

### Water‐air flux computations

The concentration gradient between water and atmosphere was computed as

(1) $${\text{dCH}}_{4}={\;\text{CH}}_{4}w-{\text{CH}}_{4}\text{eq}$$

where CH_4_eq (*μ*mol L^−1^) is the concentration in water expected for atmospheric equilibrium. CH_4_eq is computed from atmospheric pressure, CH_4_ partial pressure in air (pCH_4_), and field water temperature by Henrýs law. CH_4_ flux (F [*μ*mol m^−2^ h^−1^]) was then calculated according to:

(2) $$F=k_{\text{CH}4}\times d{\text{CH}}_{4}$$

where *k*_CH4_ is the vertical gas transfer velocity of CH_4_ (cm h^−1^) at the respective water temperature.k_CH4_ was computed from:

(3) $$k_{\text{CH}4}=k_{600}\times {\left({\text{Sc}}_{\text{CH}4}/600\right)}^{–n}$$

where *k*_600_ is the gas transfer velocity normalized to a Schmidt number of 600 and Sc_CH4_ is the Schmidt number of CH_4_ at the temperature measured in the field (Wanninkhof 2014). We used *n* = 0.67 for wind speeds in 10 m height *u*_10_ < 3.7 m s^−1^ and water velocities *v* < 30 cm s^−1^. Whenever either *u*_10_ or *v* exceeded the threshold, *n* = 0.5 was applied.

We predicted *k*_600_ (cm h^−1^) based on an additive model developed from CO_2_ data by (Borges et al. 2004):

(4) $$k_{600}\;=\;1.0\;+\;1.719v^{0.5}h^{-0.5}\;+\;2.58u_{10}$$

from flow velocity *v* (cm s^−1^), water depth *h* (m), and wind speed at 10 m height *u*_10_ (m s^−1^). The wind speed was obtained from ZAMG measured at nearby meteorological stations. Notably, Eq. (4) takes into account both hydrological conditions and wind as controls on gas transfer velocity, which contrasts classical approaches (Cole and Caraco 1998; Crusius and Wanninkhof 2003; Juutinen et al. 2009), but is considered vital for dynamic floodplain water bodies (Alin et al. 2011). For the completely disconnected station (no flow, sheltered from wind) we used a k_600_ of 2.13 (cm h^−1^) proposed for small, temperate, wind‐sheltered lakes by Cole et al. (2010).

To calculate the total CH_4_ evasion from the whole area (tCH_4_) (kmol h^−1^), the floodplain section belonging to each sampled water body was delineated based on the average connection to the Danube (Reckendorfer and Steel 2004; Tritthart et al. 2011) (Fig. 1a). The total area covered with water during distinct hydrological phases was calculated (Fig. 1b). Then, based on daily mean discharge, the following time periods were established: “mean water,” “1‐year flood,” and “100‐ year flood.” Afterwards, each CH_4_ flux datapoint was assigned to one of these categories, in each year, respectively. Assuming that our investigation period covered most of the hydrological situations, tCH_4_ for each section and for the whole river‐floodplain was determined by multiplying the average CH_4_ flux within one time period by the aquatic surface area of the respective section. We then scaled up the CH_4_ flux to obtain the total, annual evasion of tCH_4_ (kmol yr^−1^) from the whole river‐floodplain (Lobau and Regelsbrunn) for each year.

### Statistical analyses

Statistical analyses were performed with R 3.1.2 (R Core Team 2014), using the package gam (Hastie 2015). Analytical replicates (*n* = 1–3) were averaged for each site and date prior to any statistical analysis to avoid pseudoreplication. To analyze the effects of the 1‐yr and 100‐yr floods on concentrations and fluxes, we computed bootstrap‐confidence intervals (percentile method, [Manly 2006]) for the differences between pre‐flood and flood phases at the various stations. To analyze effects of hydrological isolation on CH_4_ data, we tested for differences among individual sites and accounted for temporal autocorrelation by a cubic spline smoother in general additive models (Hastie and Tibshirani 1990). To avoid variance heterogeneity, we used log‐transformed CH_4_ concentration or flux as individual responses. In these models, “site” is considered an ordered factor due to differing degrees of hydrological isolation (see above). We also included an interaction between the temporal smoothing term and site to investigate differences in temporal dynamics across sites.

## Results

### Hydrological conditions in the river‐floodplain system

**Figure 2:**
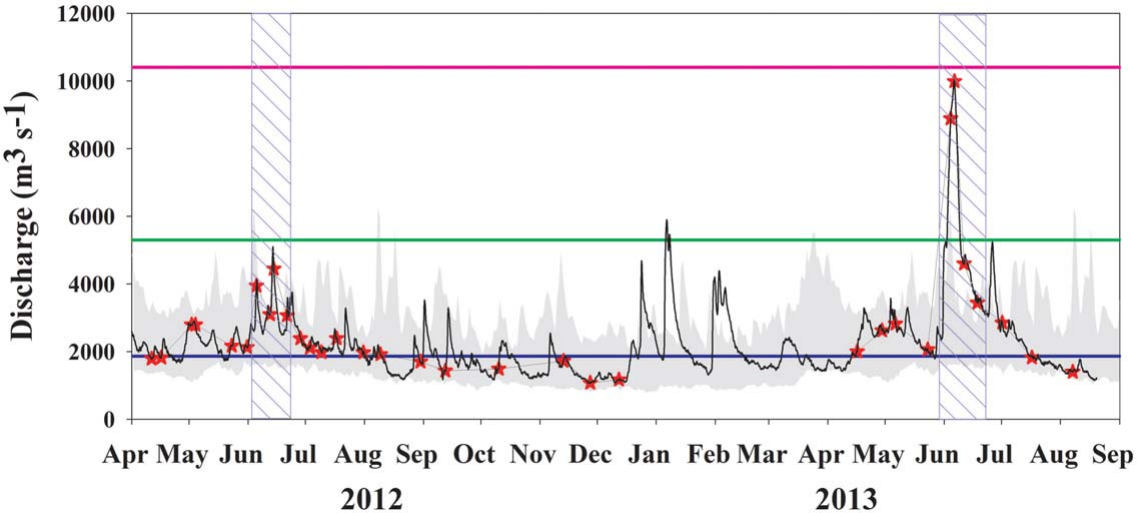
Discharge of the main channel of the Danube during the investigation period. Grey area indicates the range between the 10th and the 90th percentile of the daily mean discharge (1996–2012). Stars indicate sampling events. Dashed area indicates flooding events during the investigation period. Blue line indicates Danube discharge for mean water, green line 1‐yr flood and pink line 100‐yr flood.

Hydrological conditions in the Danube (and in the floodplain) differed markedly between the two years. In 2012, the main channel discharge ranged from 1061 to 5101 m^3^ s^−1^ (Fig. 2), with a typical 1‐yr flood in June. According to the gradient of connectivity, the dynamic stations were connected 82% (station C) and 65% (station D) of the sampling events. The semi‐isolated stations (E and F) were connected during the flood only, yet without flowing water conditions in 27% and 5% of the sampling events, respectively. In 2013, the Danube discharge ranged between 1338 m^3^ s^−1^ and 10,041 m^3^ s^−1^ (Fig. 2). The discharge maximum in early June corresponded to a 100‐yr flood and resulted in flowing water conditions at the dynamic and semi‐isolated stations. Despite the large differences in peak flow, surface connectivity in 2013 was similar to 2012. The surface connection with the Danube in dynamic stations occurred in 90% (B, C) and 65% (D), while in stations E and F it was established in 55% and 18% of the sampling events, respectively. A surface connection between the Danube and station G was never established.

### Concentrations of CH_4_ across the river‐floodplain system

**Figure 3:**
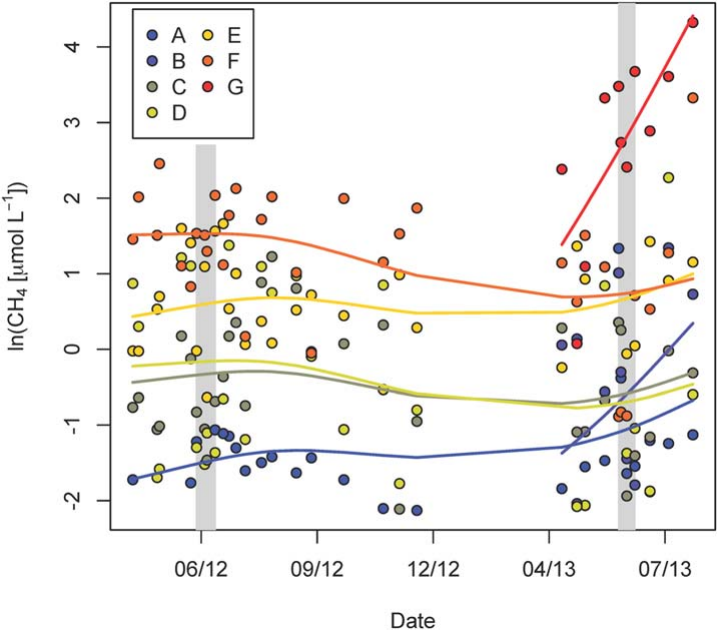
CH_4_ concentrations at the 7 investigated sites differing in hydrological isolation (surface connectivity with the Danube) across the whole study period. The color gradient indicates increasing hydrological isolation (from blue toward red). Lines are cubic spline smoothers of time resulting from a general additive model with an interaction term between (smoothed) time and site. Note converging trends of average CH_4_ concentration in the second year, that is, less spatial variation across the continuously sampled subset of sites in the year with the 100‐yr flood. For stations B and G data was only available for 2013. These two stations showed a strong increase in CH_4_ concentrations over the short period of sampling in 2013.

At all sampling events, river‐floodplain waters were supersaturated with CH_4_. Atmospheric partial pressures of CH_4_ were low (1.70–7.46 ppm) and concentrations of CH_4_ in water were 29–22,281 times higher than expected theoretically for an equilibrium with the atmosphere. Notably, concentrations showed distinct spatial variation, with clear differences among stations depending on connectivity (Fig. 3). Overall, the mean CH_4_ concentrations in water were lowest in the Danube main channel and increased with decreasing connectivity of floodplain waters (Table 1). The water in the completely isolated station (G, data for 2013 only) was exceptionally oversaturated; reaching extreme values 22,281 times in excess of the atmospheric equilibrium. There was clear temporal autocorrelation as shown by a significant smoothing term for CH_4_ on time ( $R_{\text{adj}}^{2}$=0.59, deviance explained=61.7%, *p* < 0.05, *n* = 177). Also, we identified a significant interaction between the temporal smoothing term and site, pointing to differences in CH_4_ dynamics among sites. Notably, excluding the sites measured only in the second year, this interaction can be graphically recognized by converging trends of average CH_4_ concentration in the second year, that is, less spatial variation across this subset of sites in the year with the 100‐yr flood (Fig. 3). The temperature proved to be a driving force for high methane concentrations here, resulting in a significant correlation between CH_4_ concentrations in station G and temperature (*r* = 0.62, *p* < 0.05). Also for dynamic stations, some dependency on temperature was recorded (*r* = 0.34, *p* < 0.01); at other stations no such correlation was observed.

**Table 1:** Average aquatic CH_4_ concentration (*μ*mol L^−1^) in the main channel of the Danube, dynamic, semi‐isolated and isolated stations during all sampling events.

Danube main channel	Dynamic stations	Semi‐isolated stations	Isolated station
0.41 (0.12–3.80)	1.51 (0.12–9.70)	3.58 (0.41–27.85)	24.70 (1.08–75.53)

In parentheses: min−max

**Figure 4:**
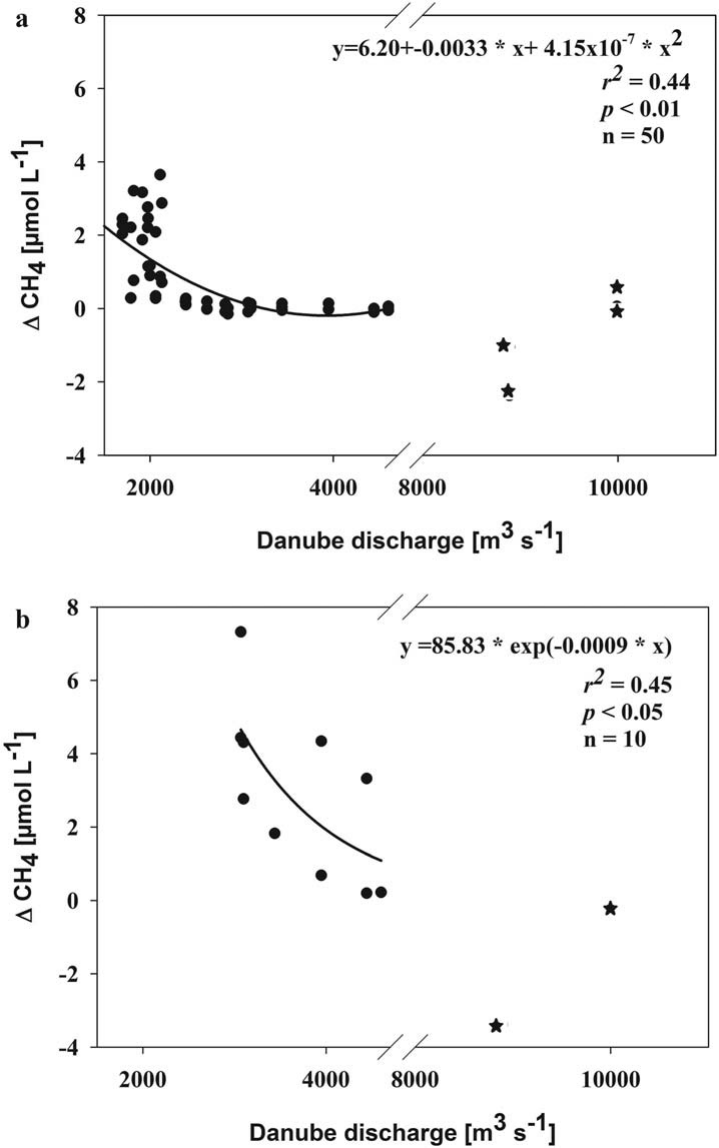
Relationships between main channel discharge and river‐to‐floodplain differences in CH_4_ concentrations in dynamic (**a**) and semi‐isolated stations (**b**) during connection with the main channel. Stars indicate samples taken during the onset of the 100‐yr flood event.

We calculated the differences in CH_4_ concentrations (ΔCH_4_) between floodplain sites and the main channel for all sampling dates during times of surface connection with the Danube. Both revealed significant inverse relationships with the Danube discharge (Fig. 4a,b). ΔCH_4_ was much larger at low discharge and decreased markedly during times of higher flow (including the 1‐yr flood). During the 100‐yr flood, the differences between the floodplain and the main channel were either very small or showed negative values, indicating considerable increase in the CH_4_ concentration in the main channel and concurrent decrease in the floodplain (Fig. 4a,b).

### CH_4_ fluxes across the river‐floodplain system—effect of flooding

**Figure 5:**
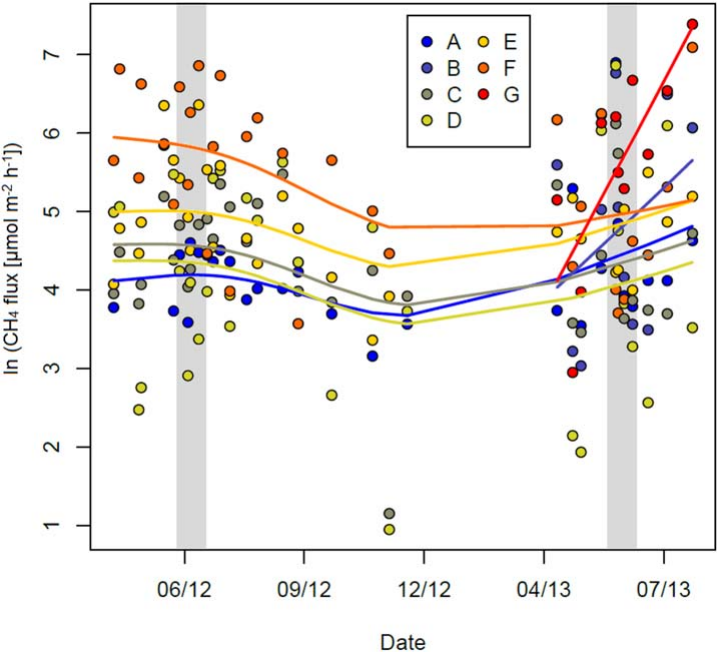
CH_4_ fluxes at the 7 investigated sites differing in hydrological isolation (surface connectivity with the Danube) across the whole study period. The color gradient indicates increasing hydrological isolation (from blue toward red). Lines are cubic spline smoothers of time resulting from a general additive model with an interaction term between (smoothed) time and site. Note converging trends of average CH_4_ concentration in the second year, that is, less spatial variation across the continuously sampled subset of sites in the year with the 100‐yr flood. For stations B and G data were only available for 2013. These two stations showed a strong increase in CH_4_ fluxes over the short period of sampling in 2013.

Overall, methane evasion differed markedly along the connectivity gradient from the main channel, through dynamic, semi‐isolated to isolated stations and revealed distinct spatial variation with clear differences among stations (Fig. 5). The lowest average methane fluxes were observed in the Danube main channel (Table 2), and the highest average values always occurred in floodplain waters; the only exception was the 100‐yr flood in the main channel of the Danube (Table 2). Furthermore, similar to CH_4_ concentration data, differences in the dynamics of CH_4_ flux among the sites were indicated by a significant interaction between the temporal smoothing term and site ( $R_{\text{adj}}^{2}$ = 0.17, deviance explained=22.5%, *p* < 0.05, *n* = 181).

**Table 2:** Average CH_4_ flux (*μ*mol m^−2^ h^−1^) from the main channel of the Danube, dynamic, semi‐isolated and isolated stations during different hydrological phases.

	Danube	Dynamic stations	Semi‐isolated stations	Isolated station
Spring pre‐ flood (both years)	72.2 (34.7–199.1)	116.7 (6.9–417.9)	304.1 (58.8–913.6)	175.9 (19.1–458.8)
1‐yr flood	77.4 (36.2–99.7)	69.5 (18.4–125.8)	430.9 (90.5–952.5)	no data
100‐yr flood	303.2 (44.3–990.2)	260.1 (26.6–955.6)	74.1 (40.7–152.8)	432.8 (198.7–791.1)
After flood (both years)	63.3 (23.5–102.5)	138.1 (2.6–661.2)	228.7 (28.8–1203.2)	871.9 (307.9–1616.2)

In parentheses: min‐max.

**Figure 6:**
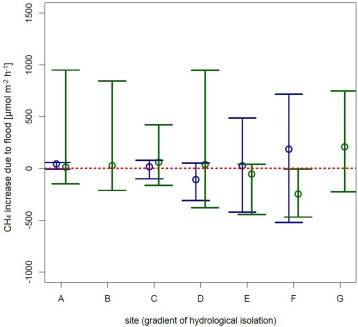
95% bootstrap confidence intervals (computed from percentiles) for the flood‐driven CH_4_ flux increase (differences *flood* minus *pre*‐*flood*) at the various sites ordered along hydrological isolation (surface connectivity) in 2012 (blue) and 2013 (green). Non‐overlap of confidence intervals with the zero line indicates a significant difference between hydrological phases. Similarly, non‐overlap among pairs of confidence intervals indicates a significant difference between pairs of sites or years. The means of analytical replicates were used for bootstrapping.

Flooding had variable impact on CH_4_ evasion from the various river‐floodplain stations. The CH_4_ flux from the Danube main channel and the dynamic stations was higher during the 100‐yr flood (Table 2) but due to high variability the difference to pre‐flood conditions was not significant (Fig. 6). The 1‐yr flood resulted in lower fluxes in dynamic floodplain stations, yet also these could not be identified as significantly different to spring‐pre flood conditions (Fig. 6). At semi‐isolated stations a significant decrease of CH_4_ evasion occurred during the 100‐yr flood (Fig. 6). At the least connected station (G), the flood had no significant effect on CH_4_ flux. No significant correlation between CH_4_ fluxes and temperature was noted except semi‐isolated (*r* = 0.26, *p* < 0.05) and isolated station (*r* = 0.70, *p* < 0.05).

### Floodplain waters vs. main channel as important sources of methane

**Figure 7:**
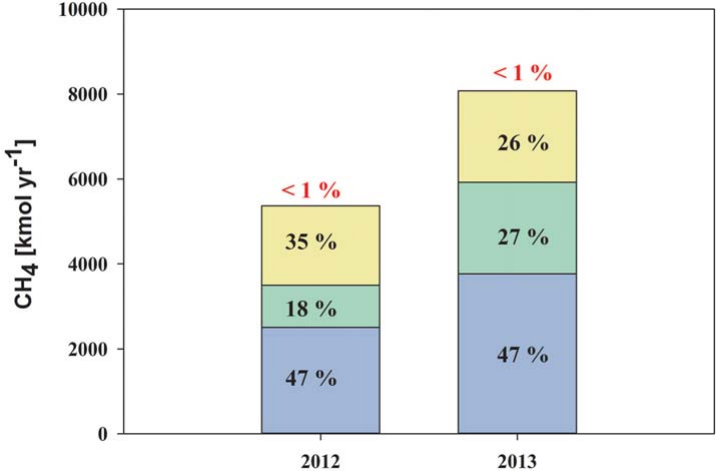
Total evasion of CH_4_ (kmol yr^−1^) from the entire river‐floodplain (Regelsbrunn and Lobau) in 2012 and 2013 and relative contribution (%) of different sections: Danube (blue), dynamic (green), semi‐isolated (yellow), isolated (red).

Based on the evasion rates and the flooded area of the two river‐floodplain systems, we evaluated the share of floodplain evasion of CH_4_ (mol h^−1^) compared to the entire river‐floodplain system (Regelsbrunn + Lobau + main channel). Overall, our results revealed that during both years, floodplain waters (dynamic and semi‐isolated) accounted for 53% (in 2012 and in 2013) of the CH_4_ evasion from the investigated river‐floodplain system (Fig. 7). The contributions of the isolated station (G) were negligible due to its small surface area.

Most of the CH_4_ (mol h^−1^) was lost during the 100‐yr flood (Table 3). The highest evasion was estimated from the water surface of the Danube stretch, but high losses were also recorded from the regulated floodplain of Lobau and restored section at Regelsbrunn (Table 3). Our results also revealed that 34% more CH_4_ (kmol yr^−1^) were emitted from the entire river‐floodplain during the year with the 100‐yr flood (Fig. 7).

**Table 3:** Average total fluxes of CH_4_ (mol h^−1^) from the Danube main channel stretch of the investigated area, Regelsbrunn (restored) and Lobau (regulated) floodplains during different hydrological phases.

	Spring pre‐flood		1‐yr flood		100‐yr flood		After flood	
	Total flux [mol h^−1^]	Area	Total flux [mol h^−1^]	Area	Total flux [mol h^−1^]	Area	Total flux [mol h^−1^]	Area
		[km^2^]		[km^2^]		[km^2^]		[km^2^]
Danube	341.37	4.87	466.46	6.16	2196.20	7.24	296.43	4.87
main channel								
Regelsbrunn	63.59	0.67	229.99	2.43	1039.91	4.35	121.36	0.67
Lobau	284.25	1.21	545.80	2.02	1935.67	14.96	201.74	1.21

## Discussion

### CH_4_ concentration in river‐floodplain sections

High spatial and temporal variability in aquatic CH_4_ concentrations is characteristic for wetlands (Bloom et al. 2012), rivers (Shelley et al. 2014) as well as for floodplains (Crill et al. 1988). In this study, methane concentrations also covered a broad range (Table 1) (Fig. 3); the main channel had lower values (29–1206 times higher than atmospheric equilibrium), while semi‐isolated and isolated floodplain waters were highly supersaturated with CH_4_ (32–22,281 times higher than atmospheric equilibrium). Hence, floodplain waters, as well as the main channel of the Danube, were always sources of CH_4_ to the atmosphere. Average aquatic methane concentrations in this study resembled the concentrations reported for the Brazilian Amazon (Belger et al. 2011) rather than the lower concentrations for temperate lowland rivers (Sanders et al. 2007; Trimmer et al. 2009). The elevated aquatic methane concentrations are likely caused by in‐situ methanogenesis, which can be considerably high in riverbed sediments (Shelley et al. 2015). The activity of methanogens is affected by temperature (Fu et al. 2015; Tveit et al. 2015), and methane formation is apparently sensitive to temperature increase (Duc et al. 2010). In our study, temperature was the strongest controlling mechanism for CH_4_ concentration at the completely disconnected station (G). However, the temperature‐dependency was less pronounced in other stations, where methane concentrations could also be influenced by other factors such as availability of organic carbon (Christensen et al. 2003; Liu et al. 2011). The quantity and quality of organic substrates delivered to the river‐floodplain are highly variable and fluctuate substantially, from mostly allochthonous material during the flood (Sieczko and Peduzzi 2014) to autochthonous material during the post‐flood period (Sieczko et al. 2015). The elevated sedimentation of organic matter delivered with a spate could also be responsible for enhanced activity of methanogens during the post‐flood period (Van Huissteden et al. 2005; Gatland et al. 2014), especially at semi‐isolated stations. Hence, beside fluctuations in methanogen activity, spatiotemporal variation in aquatic CH_4_ concentration across the river‐floodplain system can be purely physically induced by hydrological fluctuations affecting gas exchange (Pulliam and Meyer 1992). This includes flooding events, which may alter CH_4_ concentrations substantially by flushing otherwise hydrologically isolated habitats. In our study, the highest differences in concentrations between floodplain waters and the Danube were noted when the river flow was lower (Fig. 4a,b). In agreement with Pulliam and Meyer (1992), our results indicate that dissolved CH_4_ concentrations in floodplain waters are driven by changes of main channel discharge. This suggests that surface connection between the floodplain and the river may be an important factor controlling CH_4_ concentrations in the main channel (Richey et al. 1988). In fact, due to its much higher gas exchange efficiency the river main channel can act as an “exhaust pipe” of the entire river‐floodplain system, responsible for evasion of CH_4_ produced in floodplain waters. This mechanism have been especially important during the 100‐yr flood when floodplain CH_4_ concentrations decreased due to dilution, while the main channel CH_4_ concentration increased (Fig. 4a,b) at the same time. Hence, a pulse of methane flushed from the slow‐flowing floodplain into the river could be responsible for elevated CH_4_ in the main channel. This suggests that most of the river CH_4_ has originated in the supersaturated waters adjacent to the main channel and suggests that river carbon delivery was driven by surface floodwater supply (Abril et al. 2014; Gatland et al. 2014). Additionally, the high CH_4_ peak during the 100‐yr flood could also be associated with run‐off from upstream forest and agricultural soils (Angelis and Lilley 1987).

### Importance of floods: Implications of increased flooding frequency for methane evasion

High interannual variations of CH_4_ emissions, characteristic for river‐floodplains, point to the importance of these systems as methane sources (Marani and Alvala 2007). Rapid hydrological changes, typical for river‐floodplains, can substantially affect evasion of greenhouse gases from temporarily inundated systems (Altor and Mitsch 2008), and flooded environments are recognized as sources of methane to the atmosphere (Belger et al. 2011). Our study shows that flooding significantly altered methane evasion. It also emphasizes the importance of spates with different magnitude for CH_4_ emission from stations with different connectivity (Fig. 6). During a high‐water period, methane evasion can be significantly higher (Devol et al. 1990), with rapid emissions occurring shortly after the onset of the flood (Boon et al. 1997). Similar to a study of Gatland et al. (2014), our findings show that the highest CH_4_ loss from the floodplain area occurred during the extreme 100‐year flood (Table 3). This suggests that an improved, detailed characterization of hydrological processes is necessary to correctly assess the CH_4_ dynamics in river‐floodplain systems (Zhu et al. 2013).

Methane emissions from wetlands have been linked to climate change (Mitsch et al. 2010). Yvon‐Durocher et al. (2014) suggested that global warming has a large effect on the relative contributions of CH_4_ to total GHG from aquatic ecosystems. Also, impacts of global change, such as increasing frequency of droughts and flooding, summarized by Lehner et al. (2006), are expected to occur in Europe. However, the effect of a shift in hydrological variability—as possibly caused by global climate change—on methane emissions still remains unknown both at tropical latitudes (Mitsch et al. 2010) and in temperate regions. Future scenarios for the Danube River predict an increase in discharge magnitude and in water temperature during spring floods (Zweimüller et al. 2008). In our study, tCH_4_ evasion (Fig. 7) was 34% higher during the year of extreme flooding compared to the hydrologically “average” year, suggesting that increased flooding frequency, increase of the flooded area will have marked consequences on overall methane emissions (Sha et al. 2011). Hence, higher emissions from river‐floodplains during extensive floods may be expected in the future. Note also that rivers can be important CH_4_ sources to the coastal ocean (Scranton and Mcshane 1991). Pavel et al. (2009), for example, observed a significant input of riverine CH_4_ into the Danube Delta during post‐flood conditions. Thus, the predicted, intensified spate occurrence in the Danube may also have consequences for the carbon cycle in the Danube Delta and the coastal Black Sea.

### Methane evasion from floodplains with different levels of human impact

The global emissions of methane from tropical wetlands (Smith et al. 2000) and from temperate regions are most likely underestimated and considerably uncertain (Kirschke et al. 2013). Typically, large tropical floodplains have been considered to be the most important sources of methane due to their extensive area (Bartlett and Harriss 1993). Our data (Table 2), however, show that the average diffusive fluxes from semi‐isolated and isolated stations of a temperate floodplain are similar to average methane fluxes measured over flooded tropical Amazon forest (Bartlett and Harriss 1993). Our study also shows that isolated and semi‐isolated stations, although exhibiting variability in their relative contribution (Fig. 7), were significantly stronger sources of CH_4_ to the atmosphere than the dynamic sites and the respective river stretch (Table 2). Occasionally, the flux per unit water surface area reached 94% higher values than in the main channel (Table 2). Hence, our study points to the importance of semi‐isolated and isolated floodplain waters as important sources of methane.

In general, human activities significantly affect the contribution of inland waters to the carbon cycle (Regnier et al. 2013). Due to river regulation, damming or agriculture, most of the European floodplains have been severely impacted. Today, spatially much reduced floodplain areas experience a markedly lowered frequency of connection with the main river (Buijse et al. 2002). The Danube River is regulated along 80% of its length, resulting in 81% reduction of floodplain areas in the entire river basin (Günther‐Diringer 2001). Our findings emphasize that most of the CH_4_ emissions originated from human‐altered, semi‐isolated floodplain waters. Approximately 47% of CH_4_ emission occurred from the main river stretch (Fig. 7). The investigated isolated station, due to its small area, did not contribute substantially to the total CH_4_ loss from the whole river‐floodplain. High CH_4_ fluxes from this isolated station (Table 2, Fig. 5), however, suggest that ongoing, complete disconnection of floodplain sections due to river regulation will produce more areas with such water bodies, thus increasing their contribution to the overall CH_4_ budget. Our findings indicate that human‐induced disconnection of floodplain waters from the river may have significant consequences for the overall methane budget and emphasize the importance of floodplain waters as sources of greenhouse gases. River‐floodplains—beside small, dynamic streams and rivers (Benstead and Leigh 2012)—also apparently play an important role in global greenhouse gas fluxes.

Here, we focused only on diffusive fluxes; the ebullition, plant‐mediated flux or recently reported microbubble flux (Prairie and Del Giorgio 2013) have not been investigated. Especially ebullition could be an important component of the total methane flux in floodplain waters (Smith et al. 2000; Ringeval et al. 2014). In rivers, diffusive flux is usually the main component of the total CH_4_ emission, but ebullition hotspots may contribute substantially (up to 50%) to the total flux (Sawakuchi et al. 2014). The ebullition rates are also temperature‐dependent (Wilkinson et al. 2015) and may thus play an increasing role in the total CH_4_ flux in the context of global warming. Because ebullition can be the dominant mechanism in floodplains (Devol et al. 1990) and may exceed diffusive fluxes several‐fold or even by orders of magnitude in some systems, we consider this study's estimate as conservative for the contribution of floodplain waters to the overall river‐floodplain methane emission. Nevertheless, in line with other studies, our work stresses the importance of CH_4_ emissions from temperate river‐floodplain systems with different levels of human impact. Temperate river‐floodplain systems should be considered in global budgets of CH_4_ emission.

## References

[lno10346-bib-0001] Abril, G. , and others. 2014 Amazon River carbon dioxide outgassing fuelled by wetlands. Nature 505: 395–398. doi:10.1038/nature12797 24336199 10.1038/nature12797

[lno10346-bib-0002] Alin, S. R. , and others 2011 Physical controls on carbon dioxide transfer velocity and flux in low‐gradient river systems and implications for regional carbon budgets. J. Geophys. Res.: Biogeosci. 116 **: G01009**. doi:10.1029/2010JG001398

[lno10346-bib-0003] Altor, A. E. , and W. J. Mitsch . 2008 Pulsing hydrology, methane emissions and carbon dioxide fluxes in created marshes: A 2‐year ecosystem study. Wetlands 28: 423–438. doi:10.1672/07-98.1

[lno10346-bib-0004] Angelis, M. A. D. , and M. D. Lilley . 1987 Methane in surface waters of Oregon estuaries and rivers. Limnol. Oceanogr. 32: 716–722. doi:10.4319/lo.1987.32.3.0716

[lno10346-bib-0005] Aselmann, I. , and P. Crutzen . 1989 Global distribution of natural freshwater wetlands and rice paddies, their net primary productivity, seasonality and possible methane emissions. J. Atmos. Chem. 8: 307–358. doi:10.1007/BF00052709

[lno10346-bib-0006] Bartlett, K. B. , and R. C. Harriss . 1993 Review and assessment of methane emissions from wetlands. Chemosphere 26: 261–320. doi:10.1016/0045-6535(93)90427-7

[lno10346-bib-0007] Bastviken, D. , and others. 2010 Methane emissions from Pantanal, South America, during the low water season: Toward more comprehensive sampling. Environ. Sci. Technol. 44: 5450–5455. doi:10.1021/es1005048 20568738 10.1021/es1005048

[lno10346-bib-0008] Bastviken, D. , L. J. Tranvik , J. A. Downing , P. M. Crill , and A. Enrich‐Prast . 2011 Freshwater methane emissions offset the continental carbon sink. Science 331: 50–50. doi:10.1126/science.1196808 21212349 10.1126/science.1196808

[lno10346-bib-0009] Batson, J. , G. B. Noe , C. R. Hupp , K. W. Krauss , N. B. Rybicki , and E. R. Schenk . 2015 Soil greenhouse gas emissions and carbon budgeting in a short‐hydroperiod floodplain wetland. J. Geophys. Res.: Biogeosci. 120: 77–95. doi:10.1002/2014JG002817

[lno10346-bib-0010] Battin, T. J. , and others. 2008 Biophysical controls on organic carbon fluxes in fluvial networks. Nat. Geosci. 1: 95–100. doi:10.1038/ngeo101

[lno10346-bib-0011] Belger, L. , B. R. Forsberg , and J. M. Melack . 2011 Carbon dioxide and methane emissions from interfluvial wetlands in the upper Negro River basin, Brazil. Biogeochemistry 105: 171–183. doi:10.1007/s10533-010-9536-0

[lno10346-bib-0012] Benstead, J. P. , and D. S. Leigh . 2012 An expanded role for river networks. Nat. Geosci. 5: 678–679. doi:10.1038/ngeo1593

[lno10346-bib-0013] Bloom, A. , P. Palmer , A. Fraser , and D. Reay . 2012 Seasonal variability of tropical wetland CH 4 emissions: The role of the methanogen‐available carbon pool. Biogeosciences 9: 2821–2830. doi:10.5194/bg-9-2821-2012

[lno10346-bib-0014] Boon, P. I. , A. Mitchell , and K. Lee . 1997 Effects of wetting and drying on methane emissions from ephemeral floodplain wetlands in south‐eastern Australia. Hydrobiologia 357: 73–87. doi:10.1023/A:1003126601466

[lno10346-bib-0015] Borges, A. V. , and others 2004 Variability of the gas transfer velocity of CO_2_ in a macrotidal estuary (the Scheldt). Estuaries 27: 593–603. doi:10.1007/BF02907647

[lno10346-bib-0016] Buijse, A. D. , and others 2002 Restoration strategies for river floodplains along large lowland rivers in Europe. Freshwater Biol. 47: 889–907. doi:10.1046/j.1365-2427.2002.00915.x

[lno10346-bib-0017] Christensen, T. R. , and others. 2003 Factors controlling large scale variations in methane emissions from wetlands. Geophys. Res. Lett. 30: 1414. doi:10.1029/2002GL016848

[lno10346-bib-0018] Cole, J. J. , and N. F. Caraco . 1998 Atmospheric exchange of carbon dioxide in a low‐wind oligotrophic lake measured by the addition of SF6. Limnol. Oceanogr. 43: 647–656. doi:10.4319/lo.1998.43.4.0647

[lno10346-bib-0019] Cole, J. J. , D. L. Bade , D. Bastviken , M. L. Pace , and M. Van De Bogert . 2010 Multiple approaches to estimating air‐water gas exchange in small lakes. Limnol. Oceanogr.: Methods 8: 285–293. doi:10.4319/lom.2010.8.285

[lno10346-bib-0020] Crill, P. , and others. 1988 Tropospheric methane from an Amazonian floodplain lake. J. Geophys. Res. 93: 1564–1570. doi: 10.1029/JD093iD02p01564

[lno10346-bib-0021] Crusius, J. , and R. Wanninkhof . 2003 Gas transfer velocities measured at low wind speed over a lake. Limnol. Oceanogr. 48: 1010–1017. doi:10.4319/lo.2003.48.3.1010

[lno10346-bib-0022] Denman, K. L. , and others. 2007 Couplings between changes in the climate system and biogeochemistry. Clim. Change 2007: 541–584.

[lno10346-bib-0023] Devol, A. H. , J. E. Richey , B. R. Forsberg , and L. A. Martinelli . 1990 Seasonal dynamics in methane emissions from the Amazon River floodplain to the troposphere. J. Geophys. Res.: Atmos. 95: 16417–16426. doi:10.1029/JD095iD10p16417

[lno10346-bib-0024] Downing, J. A. , and others. 2012 Global abundance and size distribution of streams and rivers. Inland Waters 2: 229–236. doi:10.5268/IW-2.4.502

[lno10346-bib-0025] Duc, N. T. , P. Crill , and D. Bastviken . 2010 Implications of temperature and sediment characteristics on methane formation and oxidation in lake sediments. Biogeochemistry 100: 185–196. doi:10.1007/s10533-010-9415-8

[lno10346-bib-0026] Frolking, S. , and others. 2011 Peatlands in the Earth's 21st century climate system. Environ. Rev. 19: 371–396. doi:10.1139/a11-014

[lno10346-bib-0027] Fu, L. , T. Song , and Y. Lu . 2015 Snapshot of methanogen sensitivity to temperature in Zoige wetland from Tibetan plateau. Front. Microbiol. 6: 131. doi:10.3389/fmicb.2015.00131 25745422 10.3389/fmicb.2015.00131 PMC4333864

[lno10346-bib-0028] Gabriel, H. , A. P. Blaschke , R. Taschke , and E. Mayr . 2015. Gewässervernetzung (Neue) Donau – Untere Lobau (Nationalpark Donau‐Auen).

[lno10346-bib-0029] Gatland, J. R. , I. R. Santos , D. T. Maher , T. Duncan , and D. V. Erler . 2014 Carbon dioxide and methane emissions from an artificially drained coastal wetland during a flood: Implications for wetland global warming potential. J. Geophys. Res.: Biogeosci. 119: 1698–1716. doi:10.1002/2013JG002544

[lno10346-bib-0128] Griebler, C. , and Mösslacher, F. 2003 Grundwassereine–ökosystemare Betrachtung. Grundwasserökologie (eds Griebler C, Mösslacher F), 253–310.

[lno10346-bib-0030] Günther‐Diringer, D. 2001. Evaluation of wetlands and floodplain areas in the Danube River basin, p. 91. River restoration in Europe.

[lno10346-bib-0031] Hastie, T. 2015 gam: Generalized additive models. Rpackage version **1.12**.

[lno10346-bib-0032] Hastie, T. J. , and R. J. Tibshirani . 1990 Generalized additive models. Chapman & Hall/CRC, Boca Raton (1990).

[lno10346-bib-0033] Heiler, G. , T. Hein , and F. Schiemer . 1995 Hydrological connectivity and flood pulses as the central aspects for the integrity of a river‐floodplain system. Reg. Rivers Res. Manage. 11: 351–361. doi:10.1002/rrr.3450110309

[lno10346-bib-0034] Hein, T. , and others. 2016 Current status and restoration options for floodplains along the Danube River. Sci. Total Environ. 543: 778–790. doi:10.1016/j.scitotenv.2015.09.073 26475242 10.1016/j.scitotenv.2015.09.073

[lno10346-bib-0035] Hohensinner, S. , and others. 2013 Changes in water and land: The reconstructed Viennese riverscape from 1500 to the present. Water History 5: 145–172. doi:10.1007/s12685-013-0074-2 27069520 10.1007/s12685-013-0074-2 PMC4811290

[lno10346-bib-0036] Juutinen, S. , and others. 2009 Methane dynamics in different boreal lake types. Biogeosciences 6: 209–223. doi:10.5194/bg-6-209-2009

[lno10346-bib-0037] Kirschke, S. , and others. 2013 Three decades of global methane sources and sinks. Nat. Geosci. 6: 813–823. doi:10.1038/ngeo1955

[lno10346-bib-0038] Klimo, E. , and H. Hager . 2001 The floodplain forests in Europe: Current situations and perspectives. European Forest Institute Research Report Number 10. Brill, Leiden, The Netherlands.

[lno10346-bib-0039] Lehner, B. , P. Döll , J. Alcamo , T. Henrichs , and F. Kaspar . 2006 Estimating the impact of global change on flood and drought risks in Europe: a continental, integrated analysis. Clim. Change 75: 273–299. doi:10.1007/s10584-006-6338-4

[lno10346-bib-0040] Liu, D. , W. Ding , Z. Jia , and Z. Cai . 2011 Relation between methanogenic archaea and methane production potential in selected natural wetland ecosystems across China. Biogeosciences 8: 329–338. doi:10.5194/bg-8-329-2011

[lno10346-bib-0041] Manly, B. F. 2006 Randomization, bootstrap and Monte Carlo methods in biology. Chapman & Hall/CRC.

[lno10346-bib-0042] Marani, L. , and P. Alvala . 2007 Methane emissions from lakes and floodplains in Pantanal, Brazil. Atmospheric Environment 41: 1627–1633. doi:10.1016/j.atmosenv.2006.10.046

[lno10346-bib-0043] Melack, J. M. , and others. 2004 Regionalization of methane emissions in the Amazon Basin with microwave remote sensing. Global Change Biol. 10: 530–544. doi:10.1111/j.1365-2486.2004.00763.x

[lno10346-bib-0044] Mitsch, W. J. , A. Nahlik , P. Wolski , B. Bernal , L. Zhang , and L. Ramberg . 2010 Tropical wetlands: Seasonal hydrologic pulsing, carbon sequestration, and methane emissions. Wetlands Ecol. Manage. 18: 573–586. doi:10.1007/s11273-009-9164-4

[lno10346-bib-0045] Otter, L. B. , and M. C. Scholes . 2000 Methane sources and sinks in a periodically flooded South African savanna. Global Biogeochem. Cycles 14: 97–111. doi:10.1029/1999GB900068

[lno10346-bib-0046] Pavel, A. , E. Durisch‐Kaiser , S. Balan , S. Radan , S. Sobek , and B. Wehrli . 2009 Sources and emission of greenhouse gases in Danube Delta lakes. Environ. Sci. Pollution Res. 16: 86–91. doi:10.1007/s11356-009-0182-9 10.1007/s11356-009-0182-9 19506929

[lno10346-bib-0047] Petrescu, A. M. R. , and others. 2015 The uncertain climate footprint of wetlands under human pressure. Proc. Natl. Acad. Sci. 112: 4594–4599. doi:10.1073/pnas.1416267112 25831506 10.1073/pnas.1416267112 PMC4403212

[lno10346-bib-0048] Prairie, Y. , and P. Del Giorgio . 2013 A new pathway of freshwater methane emissions and the putative importance of microbubbles. Inland Waters 3: 311–320. doi:10.5268/IW-3.3.542

[lno10346-bib-0049] Pulliam, W. M. , and J. L. Meyer . 1992 Methane emissions from floodplain swamps of the Ogeechee River: Long‐term patterns and effects of climate change. Biogeochemistry 15: 151–174. doi:10.1007/BF00002934

[lno10346-bib-0050] Raymond, P. A. , and others. 2013 Global carbon dioxide emissions from inland waters. Nature 503: 355–359. doi:10.1038/nature12760 24256802 10.1038/nature12760

[lno10346-bib-0150] Team, R.C. 2014 “R: A language and environment for statistical computing. R Foundation for Statistical Computing, Vienna, Austria. 2013”. ISBN 3‐900051‐07‐0.

[lno10346-bib-0051] Reckendorfer, W. , and T. Hein . 2000. Morphometrie, hydrologie und sedimentologie in der Unteren Lobau. Bericht im Rahmen des Projektes LIFE98NAT/A/005422, Nationalpark Donau‐Auen GmbH.

[lno10346-bib-0052] Reckendorfer, W. , and A. Steel . 2004. Auswirkungen der hydrologischen vernetzung zwischen fluss und au auf hydrologie, morphologie und sedimente–effects of hydrological connectivity on hydrology, morphology and sediments. na.

[lno10346-bib-0053] Reckendorfer, W. , A. Funk , C. Gschöpf , T. Hein , F. Schiemer , and S. Arnott . 2013 Aquatic ecosystem functions of an isolated floodplain and their implications for flood retention and management. J. Appl. Ecol. 50: 119–128. doi:10.1111/1365-2664.12029

[lno10346-bib-0054] Regnier, P. , and others. 2013 Anthropogenic perturbation of the carbon fluxes from land to ocean. Nat. Geosci. 6: 597–607. doi:10.1038/ngeo1830

[lno10346-bib-0055] Richey, J. E. , A. H. Devol , S. C. Wofsy , R. Victoria , and M. N. G. Riberio . 1988 Biogenic gases and the oxidation and reduction of carbon in Amazon River and floodplain waters. Limnol. Oceanogr. 33: 551–561. doi:10.4319/lo.1988.33.4.0551

[lno10346-bib-0056] Ringeval, B. , and others. 2014 Methane emissions from floodplains in the Amazon Basin: Challenges in developing a process‐based model for global applications. Biogeosciences 11: 1519–1558. doi:10.5194/bg-11-1519-2014

[lno10346-bib-0057] Saarnio, S. , W. Winiwarter , and J. Leitão . 2009 Methane release from wetlands and watercourses in Europe. Atmos. Environ. 43: 1421–1429. doi:10.1016/j.atmosenv.2008.04.007

[lno10346-bib-0058] Sander, R. 2014 Compilation of Henry's law constants, version 3.99. Atmos. Chem. Phys. Discuss. 14: 29615–30521. doi:10.5194/acpd-14-29615-2014

[lno10346-bib-0059] Sanders, I. , and others. 2007 Emission of methane from chalk streams has potential implications for agricultural practices. Freshwater Biol. 52: 1176–1186. doi:10.1111/j.1365-2427.2007.01745.x

[lno10346-bib-0060] Sawakuchi, H. O. , D. Bastviken , A. O. Sawakuchi , A. V. Krusche , M. V. Ballester , and J. E. Richey . 2014 Methane emissions from Amazonian Rivers and their contribution to the global methane budget. Global Change Biol. 20: 2829–2840. doi:10.1111/gcb.12646 10.1111/gcb.12646 24890429

[lno10346-bib-0061] Schiemer, F. , C. Baumgartner , and K. Tockner . 1999 Restoration of floodplain rivers: The Danube restoration project. Reg. Rivers Res. Manage. 15: 231–244. doi:10.1002/(SICI)1099‐1646(199901/06)15:1/3 < 231::AID‐RRR548 >3.0.CO;2‐5

[lno10346-bib-0062] Schiemer, F. , T. Hein , and P. Peduzzi . 2006 Hydrological control of system characteristics of floodplain lakes. Int. J. Ecohydrol. Hydrobiol. 6: 1–18. doi:10.1016/S1642-3593(06)70121-5

[lno10346-bib-0063] Scranton, M. I. , and K. Mcshane . 1991 Methane fluxes in the southern North Sea: The role of European rivers. Continental Shelf Res. 11: 37–52. doi:10.1016/0278-4343(91)90033-3

[lno10346-bib-0064] Sha, C. , and others. 2011 Methane emissions from freshwater riverine wetlands. Ecol. Eng. 37: 16–24. doi:10.1016/j.ecoleng.2010.07.022

[lno10346-bib-0065] Shelley, F. , J. Grey , and M. Trimmer . 2014 Widespread methanotrophic primary production in lowland chalk rivers. Proc. R. Soc. Lond. B: Biol. Sci. 281: 20132854. doi:10.1098/rspb.2013.2854 10.1098/rspb.2013.2854 PMC3996595 24695425

[lno10346-bib-0066] Shelley, F. , F. Abdullahi , J. Grey , and M. Trimmer . 2015 Microbial methane cycling in the bed of a chalk river: Oxidation has the potential to match methanogenesis enhanced by warming. Freshwater Biol. 60: 150–160. doi:10.1111/fwb.12480

[lno10346-bib-0067] Sieczko, A. , and P. Peduzzi . 2014 Origin, enzymatic response and fate of dissolved organic matter during flood and non‐flood conditions in a river‐floodplain system of the Danube (Austria). Aquat Sci 76: 115–129. doi:10.1007/s00027-013-0318-3 24415892 10.1007/s00027-013-0318-3 PMC3883529

[lno10346-bib-0068] Sieczko, A. , M. Maschek , and P. Peduzzi . 2015 Algal extracellular release in river‐floodplain dissolved organic matter: Response of extracellular enzymatic activity during a post‐flood period. Front. Microbiol. 6: 80. doi:10.3389/fmicb.2015.00080 25741326 10.3389/fmicb.2015.00080 PMC4330910

[lno10346-bib-0069] Smith, L. K. , W. M. Lewis, Jr. , J. P. Chanton , G. Cronin , and S. K. Hamilton . 2000 Methane emissions from the Orinoco River floodplain, Venezuela. Biogeochemistry 51: 113–140. doi:10.1023/A:1006443429909

[lno10346-bib-0070] Trimmer, M. , A. G. Hildrew , M. C. Jackson , J. L. Pretty , and J. Grey . 2009 Evidence for the role of methane‐derived carbon in a free‐flowing, lowland river food web. Limnol. Oceanogr. 54: 1541–1547. doi:10.4319/lo.2009.54.5.1541

[lno10346-bib-0071] Tritthart, M. , N. Welti , E. Bondar‐Kunze , G. Pinay , T. Hein , and H. Habersack . 2011 Modelling highly variable environmental factors to assess potential microbial respiration in complex floodplain landscapes. Environ. Model. Softw. 26: 1097–1111. doi:10.1016/j.envsoft.2011.04.001 10.1016/j.envsoft.2011.04.001 PMC4461192 27667961

[lno10346-bib-0072] Tveit, A. T. , T. Urich , P. Frenzel , and M. M. Svenning . 2015 Metabolic and trophic interactions modulate methane production by Arctic peat microbiota in response to warming. Proc. Natl. Acad. Sci. USA 112: E2507–E2516. doi:10.1073/pnas.1420797112 25918393 10.1073/pnas.1420797112 PMC4434766

[lno10346-bib-0073] Van Huissteden, J. , T. Maximov , and A. Dolman . 2005 High methane flux from an arctic floodplain (Indigirka lowlands, eastern Siberia). J. Geophys. Res.: Biogeosci. 110: G02002. doi:10.1029/2005JG000010

[lno10346-bib-0074] Verpoorter, C. , T. Kutser , D. A. Seekell , and L. J. Tranvik . 2014 A global inventory of lakes based on high‐resolution satellite imagery. Geophys. Res. Lett. 41: 6396–6402. doi:10.1002/2014GL060641

[lno10346-bib-0075] Wanninkhof, R. 2014 Relationship between wind speed and gas exchange over the ocean revisited. Limnol. Oceanogr.: Methods 12: 351–362. doi:10.4319/lom.2014.12.351

[lno10346-bib-0076] Wilkinson, J. , A. Maeck , Z. Alshboul , and A. Lorke . 2015 Continuous seasonal river Ebullition measurements linked to sediment methane formation. Environ. Sci. Technol. 49: 13121–13129. doi:10.1021/acs.est.5b01525 26477785 10.1021/acs.est.5b01525

[lno10346-bib-0077] Yavitt, J. B. 2010 Biogeochemistry: Cryptic wetlands. Nat. Geosci. 3: 749–750. doi:10.1038/ngeo999

[lno10346-bib-0078] Yvon‐Durocher, G. , and others. 2014 Methane fluxes show consistent temperature dependence across microbial to ecosystem scales. Nature 507: 488–491. doi:10.1038/nature13164 24670769 10.1038/nature13164

[lno10346-bib-0079] Zhu, X. , Q. Zhuang , Z. Qin , M. Glagolev , and L. Song . 2013 Estimating wetland methane emissions from the northern high latitudes from 1990 to 2009 using artificial neural networks. Global Biogeochem. Cycles 27: 592–604. doi:10.1002/gbc.20052

[lno10346-bib-0080] Zweimüller, I. , M. Zessner , and T. Hein . 2008 Effects of climate change on nitrate loads in a large river: The Austrian Danube as example. Hydrol. Process. 22: 1022–1036. doi:10.1002/hyp.7000

